# In the shadow of the laser phantom needle cross: dynamic air-plasma aperture sheds light on terahertz microscopy

**DOI:** 10.1038/s41377-022-00845-1

**Published:** 2022-05-20

**Authors:** Daniel Headland, Withawat Withayachumnankul

**Affiliations:** 1 Optoelectronics and Laser Technology Group, Department of Electronics Technology, Universidad Carlos III de Madrid, 28911 Madrid, Spain; 2 Terahertz Engineering Laboratory, School of Electrical and Electronic Engineering, The University of Adelaide, Adelaide, SA 5005 Australia

**Keywords:** Optics and photonics, Microscopy

## Abstract

Two plasma filaments crossing above the target create a subwavelength window for terahertz microscopy that excludes any subwavelength probe in vicinity.

The prospect of accessing a microscale world with terahertz waves has been attractive to researchers worldwide since the beginning of modern terahertz technology, which was spurred by ultrafast laser science^1^. Terahertz imaging with resolution in the order of microns provides great insights into carrier dynamics in semiconductors^2^, water content in biological samples^3^, and near-field interactions of artificial structures^4^, to name a few. Nonetheless, such a feat comes with great technical challenges as the spatial resolution of far-field imaging is always restricted by the diffraction limit, which is in the order of a wavelength. To put this into perspective, far-field terahertz optics at 1 THz can achieve around 300-micron resolution, or slightly less. Sample features below this scale are impressed upon evanescent fields that decay exponentially from the surface, leaving no trace available to a detector placed far away. It is therefore essential to access this information in the so-called near-field region, where the evanescent waves remain strong.

Various near-field imaging techniques existed in the optical domain long before the advent of modern terahertz technology, and fortunately, scalability holds true for electromagnetics. This has made it possible to directly adopt those existing techniques in the terahertz range, albeit with some differences in the implementation details. The move in this direction is not unprecedented, as there have been strong ties to optics since the early days of terahertz technology. One prevalent microscopic imaging approach is to introduce a physical probe near to the surface of a target, so as to collect subwavelength information in the near-field^5^. Such a probe could be in the form of a subwavelength aperture^1^, a scattering tip^6,7^, a metamaterial fiber^8^, or, recently, a terajet microsphere^9^.

Other techniques involve conversion between terahertz and optical waves to exploit the higher spatial resolution offered by shorter wavelengths. Specifically, a sample can be positioned near to a terahertz source where an emerging terahertz wave converted from an optical excitation is deeply subwavelength^10^. Common terahertz sources for this purpose include an electro-optic crystal and a photoconductive antenna. Similarly, a sample can be placed nearby a terahertz detector that is gated by an optical signal with a relatively small spot size to resolve subwavelength sample features. All of these approaches require microscale proximity of a sample to some physical structure, which is cumbersome, and could potentially damage the sample surface. To address this, an unorthodox approach known as laser terahertz emission microscopy^11^ locally converts ultrashort optical pulses to terahertz pulses via a nonlinear process within the target itself. However, although no physical structure is introduced near the target, this approach is only applicable to biased semiconductors.

In this issue of *Light*; *Science & Applications*, X. Wang and his colleagues from Capital Normal University, Beijing, and the Xian University of Technology present a demonstration of air-plasmas for terahertz near-field imaging^12^. Unlike an earlier air-plasma terahertz microscopy^13^, the sample under test is not exposed to terahertz waves generated by a plasma filament, which could cause damage. Rather, two pulsed 0.3-mJ femtosecond laser beams are focused tightly, and cross each other a few tens to hundreds of microns above the sample surface, as shown in Fig. 1. The resultant plasma filaments create a transient, subwavelength window of charges that blocks terahertz transmission. A portion of the incident terahertz beam is modulated by this dynamic subwavelength aperture before reaching and being reflected by the sample surface. The demodulated signal therefore represents the response of a highly localized portion of the surface that corresponds to the plasma window size. It should be pointed out that this proposed approach is in analogy to a dynamic aperture created by photoexcited carriers on a semiconductor plate^14^, but with air as a replacement. In this way, the authors successfully circumvent the issue of a physical structure in proximity to the sample. To validate its generality, this near-field microscopy was trialled with various types of samples.

**Fig. 1:**
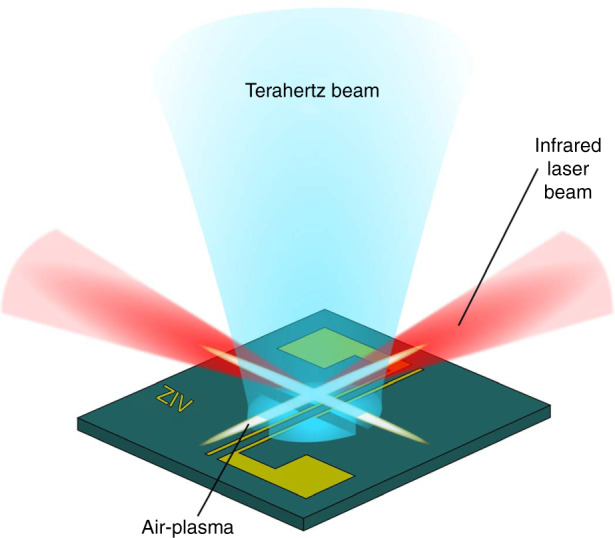
Schematic of the two crossing plasma filaments opening up a dynamic aperture for terahertz near-field scanning

At this stage, the spatial resolution is limited to half a wavelength. Nonetheless, there is room for improvement on this front to follow this pioneer work. The authors pointed out tradeoffs between the resolution, signal-to-noise ratio, and laser pulse energy that could damage the sample surface. It is anticipated that further optimization of the setup will lead to an air-plasma terahertz microscope with sub-micron resolution.

## References

[1] Hunsche S (1998). THz near-field imaging. Optical Commun..

[2] Buersgens F, Kersting R, Chen HT (2006). Terahertz microscopy of charge carriers in semiconductors. Appl. Phys. Lett..

[3] Chiu CM (2009). All-terahertz fiber-scanning near-field microscopy. Opt. Lett..

[4] Bitzer A (2009). Terahertz near-field imaging of electric and magnetic resonances of a planar metamaterial. Opt. Express.

[5] Adam AJL (2011). Review of near-field terahertz measurement methods and their applications. J. Infrared, Millim., Terahertz Waves.

[6] Huber AJ (2008). Terahertz near-field nanoscopy of mobile carriers in single semiconductor nanodevices. Nano Lett..

[7] Chen XZ (2020). THz near-field imaging of extreme subwavelength metal structures. ACS Photonics.

[8] Tuniz A (2013). Metamaterial fibres for subdiffraction imaging and focusing at terahertz frequencies over optically long distances. Nat. Commun..

[9] Pham HHN (2017). Enhancement of spatial resolution of terahertz imaging systems based on terajet generation by dielectric cube. APL Photonics.

[10] Bitzer A, Ortner A, Walther M (2010). Terahertz near-field microscopy with subwavelength spatial resolution based on photoconductive antennas. Appl. Opt..

[11] Yamashita M (2008). Noncontact inspection technique for electrical failures in semiconductor devices using a laser terahertz emission microscope. Appl. Phys. Lett..

[12] Wang X (2022). Terahertz near-field microscopy based on an air-plasma dynamic aperture. Light.: Sci. Appl..

[13] Zhao JY (2014). Terahertz imaging with sub-wavelength resolution by femtosecond laser filament in air. Sci. Rep..

[14] Chen Q (2000). Near-field terahertz imaging with a dynamic aperture. Opt. Lett..

